# Reconstruction of xenogeneic seminiferous tubule-like structures in mouse testis using cryopreserved rat testicular cells

**DOI:** 10.1016/j.isci.2026.117223

**Published:** 2026-08-13

**Authors:** Tetsuhiro Yokonishi, Blanche Capel

**Affiliations:** 1 Department of Anatomy, Kawasaki Medical School, Kurashiki, Okayama, Japan; 2 Department of Cell Biology, Duke University Medical Center, Durham, NC, USA

**Keywords:** xenogeneic spermatogonial transplantation, seminiferous tubule reconstruction, spermatogonial stem cells, spermatogonial stem cell niche, testicular niche engineering, spermatogenesis, fertility preservation

## Abstract

Spermatogonial stem cells sustain spermatogenesis within a Sertoli cell-organized niche, yet xenogeneic spermatogenic differentiation after transplantation into host mouse seminiferous tubules has been reported in only a limited number of donor species. Here, we tested whether host niche engineering could support xenogeneic spermatogenic differentiation. Immunodeficient mouse testes were preconditioned by busulfan-mediated germ cell depletion followed by intratubular benzalkonium chloride treatment to reduce Sertoli cells, and cryopreserved rat testicular cells were transplanted into the modified tubules. Donor-derived germ cells and Sertoli cells colonized the basement membrane, reconstructed seminiferous tubule-like structures, and supported advanced spermatogenic differentiation, including elongating spermatids. These findings show that frozen-thawed donor testicular cells can organize seminiferous tubule-like structures across species boundaries and provide a framework for investigating engineered testicular niches.

## Highlights

• Host niche engineering promotes xenogeneic donor cell engraftment
• Busulfan plus BC enhances seminiferous tubule-like reconstruction
• Frozen-thawed rat testicular cells organize tubule-like structures in mice
• Reconstructed xenogeneic tubules support advanced spermatid differentiation

## Graphical abstract

**Figure:**
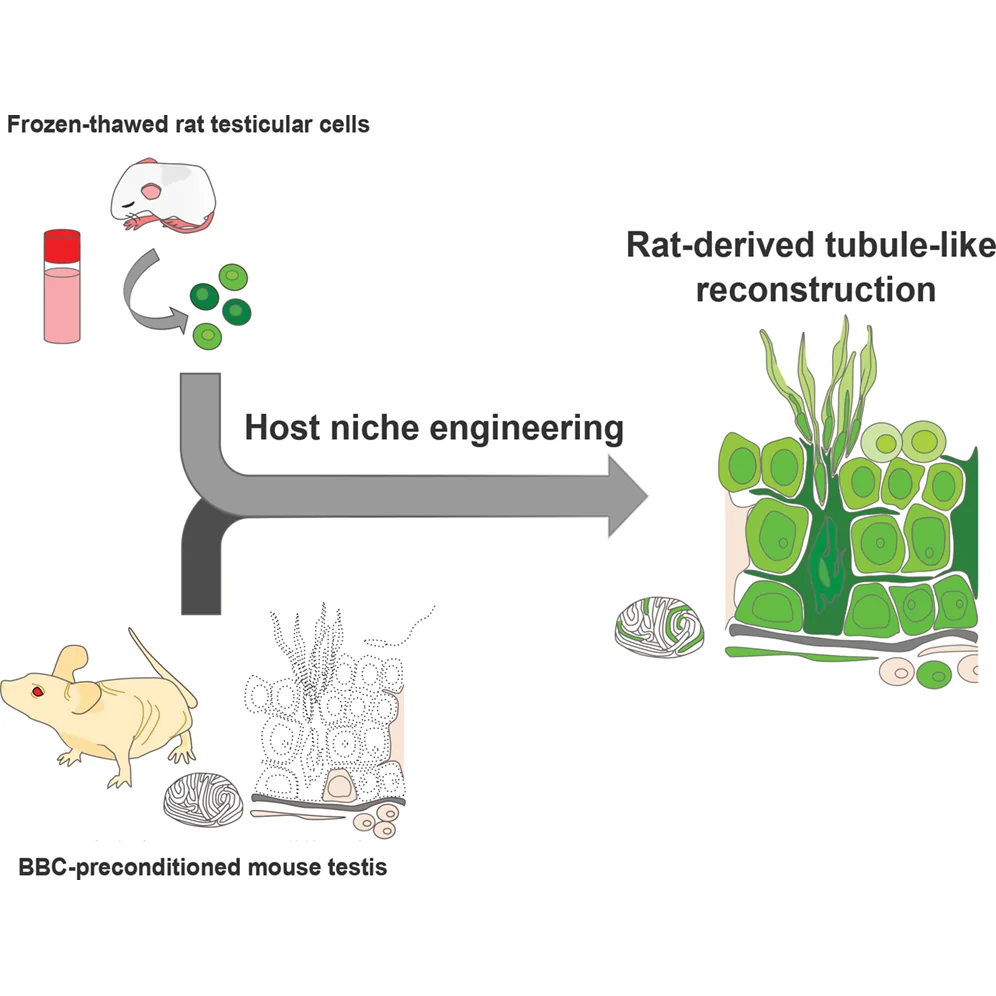

## Teaser

Biological sciences; Developmental biology

## Introduction

Since the pioneering work of Brinster and Zimmermann in the mid-1990s, spermatogonial transplantation has emerged as a transformative technique in reproductive biology and regenerative medicine.^1^ This approach enables donor spermatogonia to integrate into recipient seminiferous tubules and undergo spermatogenic differentiation. Spermatogonial transplantation has been extensively studied both as a model to investigate spermatogonial function and as a potential fertility preservation strategy, particularly for prepubertal cancer patients undergoing gonadotoxic therapies.^2^ Despite considerable advancements in autologous and allogeneic spermatogonial transplantation,^3^^,^^4^ xenogeneic transplantation—across species boundaries—has only achieved full spermatogenesis in a limited number of species.^5^^,^^6^ Successful xenogeneic spermatogonial transplantation could revolutionize reproductive medicine by enabling sperm generation from endangered species or even differentiation of human spermatogonia in surrogate animal hosts. However, substantial biological barriers remain, including immune rejection and species-specific incompatibilities between donor spermatogonia and the recipient Sertoli cells and broader spermatogonial stem cell (SSC) niche. Even in immunodeficient hosts, donor spermatogonia often fail to survive, self-renew, or differentiate, likely due to mismatched molecular signaling and inadequate microenvironmental support. Consequently, most attempts at xenogeneic spermatogonial transplantation—including those between humans and mice—have not supported complete spermatogenesis.^7^

Importantly, previous studies of xenogeneic spermatogonial transplantation have utilized the host seminiferous tubule structure and SSC niche, replacing only the SSCs. These studies did not address whether the seminiferous tubule structure itself could be reconstructed *de novo* to align with the donor SSCs. As a result, fundamental questions remain regarding whether a permissive microenvironment that supports donor-derived spermatogenic differentiation could be engineered across species boundaries.

The SSC niche is a highly regulated microenvironment primarily maintained by Sertoli cells, which orchestrate spermatogonia behavior through paracrine signals, cell-cell interactions, and metabolic support.^8^ Therefore, engineering a receptive testicular niche is essential for successful xenogeneic transplantation. To address this, we previously developed a seminiferous tubule reconstruction method involving co-transplantation of Sertoli cells and germ cells, aiming to reconstitute a functional SSC niche *in vivo*.^9^

In the present study, we build on this approach by introducing a preconditioning strategy that combines busulfan (Bus)-induced germ cell ablation with benzalkonium chloride (BC)-mediated Sertoli cell ablation. This dual treatment creates vacant seminiferous tubules in recipient testes, providing an optimized environment for donor spermatogonia and somatic cell engraftment.

To our knowledge, this is the first study to successfully reconstruct seminiferous tubule-like structures from xenogeneic donor cells, enabling advanced cross-species spermatogenic differentiation *in vivo*. This achievement overcomes long-standing barriers in niche incompatibility and represents a major step forward in reproductive tissue engineering.

The primary objectives of this study are 3-fold: (1) to evaluate the efficacy of our preconditioning strategy in depleting host germ and Sertoli cells, (2) to assess the extent of donor spermatogonial engraftment and tubule reconstruction, and (3) to characterize spermatogenic differentiation within reconstructed tubules. Together, our findings demonstrate that precise SSC niche engineering can overcome critical interspecies barriers and may provide a foundation for future studies on fertility preservation.

## Results

### Optimizing BC concentration and evaluating the effects of additional Bus preconditioning

**Figure 1:**
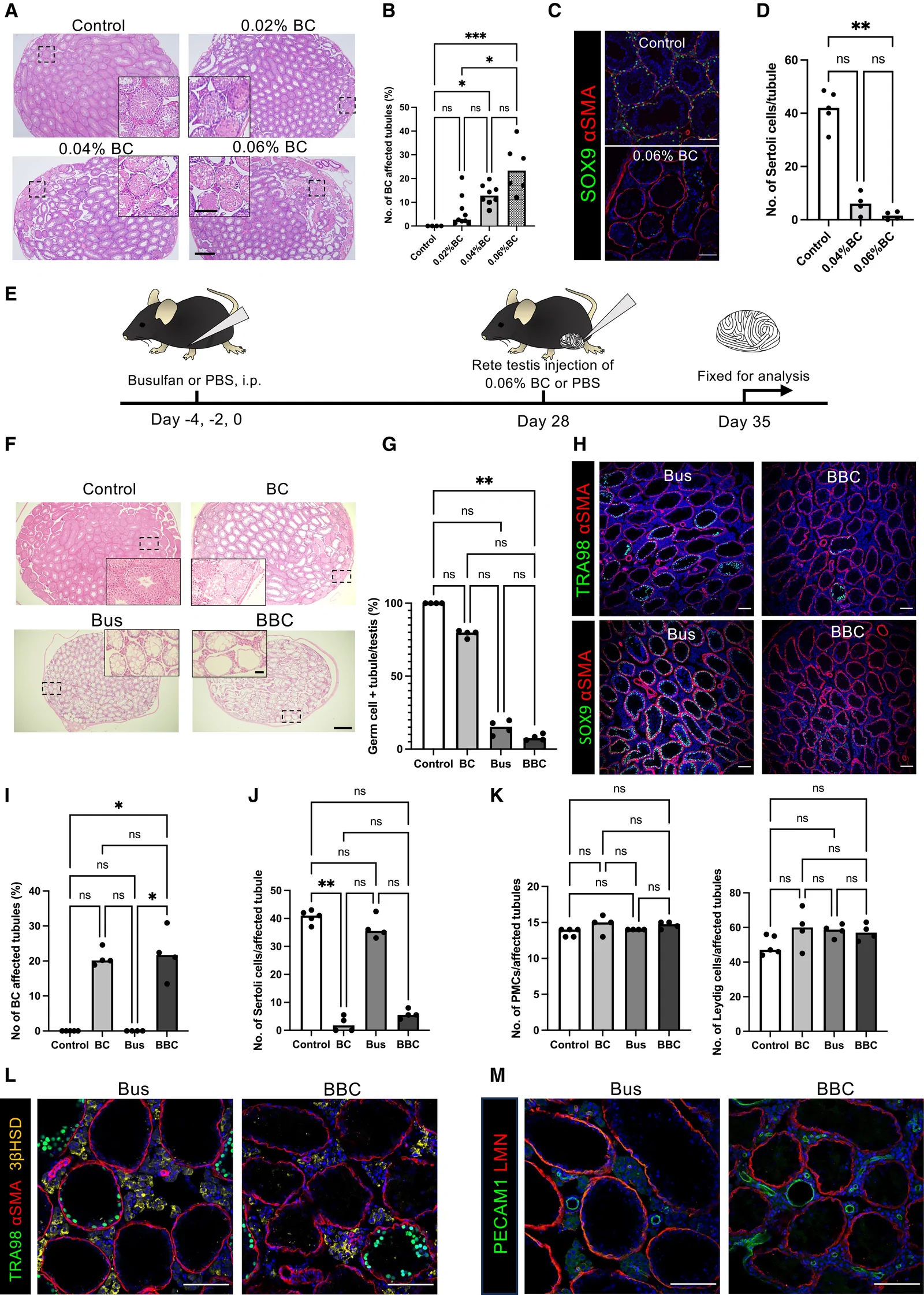
Optimization of benzalkonium chloride concentration and evaluation of combined busulfan + BC preconditioning (A) H&E-stained testis sections showing tubular damage following intratubular injection of PBS or varying benzalkonium chloride (BC) concentrations. The rectangular area outlined with a broken line is shown at higher magnification. (B) Dose-dependent reduction of SOX9^+^ Sertoli cells across BC concentrations. Bars indicate the median, and each dot represents one mouse. Four independent experiments were performed; *n* = 4, 9, 8, and 6 mice for control, 0.02% BC, 0.04% BC, and 0.06% BC groups, respectively. Asterisks indicate significant differences: ∗∗∗*p* = 0.0004, control vs. 0.06% BC; ∗*p* = 0.0217, control vs. 0.04% BC; and ∗*p* = 0.0254, 0.02% vs. 0.06% BC. (C) Immunofluorescence images of SOX9^+^ Sertoli cells (green) and αSMA^+^ peritubular myoid cells (PMCs) (red) following 0.06% BC treatment. Images in (A) and (C) are representative of four independent experiments corresponding to the quantitative analysis shown in (B). (D) Quantification of Sertoli cell numbers in affected tubules. Bars indicate the median, and each dot represents the median value obtained from three mice in one independent experiment. *n* = 5, 4, and 4 independent experiments for the control, 0.04% BC, and 0.06% BC groups, respectively. Each independent experiment included three mice per group. A significant difference was observed between the control and 0.06% BC group (∗∗*p* = 0.0066). (E) Experimental timeline for Bus and BC treatments in the non-transplantation experiment. The final intraperitoneal injection was designated as day 0. Mice received intraperitoneal injections of Bus or PBS every other day on days −4, −2, and 0. On day 28, 0.06% BC or PBS was administered by a rete testis injection, and the testes were collected and fixed 7 days later on day 35. Control mice received PBS for both treatments; the Bus group received Bus followed by PBS; the BC group received PBS followed by BC; and the BBC group received Bus followed by BC. (F) Representative histology of testes from control, Bus, BC, and BBC groups. The treatment conditions for each group are defined in (E). The rectangular area outlined with a broken line is shown at higher magnification. (G) Proportion of seminiferous tubules containing TRA98-positive cells. Bars indicate the median, and each dot represents the median value of three mice from one independent experiment. Four independent experiments were performed. ∗∗ indicates a significant difference between control and BBC groups (∗∗*p* = 0.0026). ns, not significant. (H) Immunostaining of Bus and BBC testes. Tissues were stained for TRA98 (green) and αSMA (red) (upper), and SOX9 (green) (lower) with Hoechst (blue). (I–K) Quantitative analyses of affected seminiferous tubules and testicular somatic cell populations. Bars indicate the median, and each dot represents the median value of three mice from one independent experiment. *n* = 5, 4, 4, and 4 independent experiments for the control, BC, Bus and BBC groups, respectively. (I) Proportion of seminiferous tubules affected by BC treatment. ∗ indicates a significant difference between the control and BBC groups (∗*p* = 0.0241) and between the Bus and BBC groups (∗*p* = 0.0381). (J) Number of Sertoli cells in BC-affected seminiferous tubules. A significant difference was observed between the control and BC groups (∗∗*p* = 0.0055). ns, not significant. (K) Quantification of PMCs and Leydig cells across experimental groups. (L and M) Immunostaining of interstitial cells in testes treated with Bus or BBC.(L) Tissues were stained with antibodies against TRA98 (green), αSMA (red), and 3βHSD (yellow), with Hoechst nuclear staining (blue).(M) Tissues were stained with antibodies against PECAM1 (platelet/endothelial cell adhesion molecule 1; green), and laminin (red), with Hoechst (blue). Images in (F) are representative of four independent experiments for each treatment group shown. Images in (H), (L), and (M) are representative of four independent experiments for each of the Bus and BBC groups. Scale bars: 500 μm (A and F, overview); 100 μm (A magnified images, C, H, L, and M); 50 μm (F, inset). Statistical comparisons were made using Kruskal-Wallis test followed by Dunn’s multiple-comparisons test. ∗*p* < 0.05, ∗∗*p* < 0.01, and ∗∗∗*p* < 0.001; ns, not significant.

To optimize Sertoli cell ablation, we first investigated the effects of different concentrations of BC administered via intratubular injection into adult mouse seminiferous tubules. Although our previous study demonstrated that 0.02% BC was sufficient to eliminate Sertoli cells,^9^ in the present study, this concentration failed to induce detectable ablation (Figure 1A). The discrepancy may be attributable to differences in mouse strain, age, or experimental environment, although the precise cause remains unclear. Importantly, no signs of systemic toxicity or mortality were observed across all tested concentrations. As the BC concentration increased, the proportion of SOX9^+^ Sertoli cells progressively decreased (Figures 1B and 1C), with 0.06% BC producing the most substantial Sertoli cell ablation as confirmed by immunofluorescence (Figure 1D).

We next tested whether preconditioning with Bus—an alkylating agent commonly used to eliminate endogenous spermatogonia^1^—could enhance the efficacy of BC. Mice received three intraperitoneal injections of Bus on alternating days, followed by intratubular administration of 0.06% BC 4 weeks after the final Bus injection. Testes were collected and analyzed 7 days after BC treatment (Figure 1E). Bus- and BC-treated testes exhibited marked atrophy (Figure 1F). In the BC group, germ cells marked by TRA98, an antibody recognizing germ cell nuclear antigen 1 (GCNA1), were partially depleted (Figure 1G). Consistent with our previous report, BC treatment primarily targets Sertoli cells, leading to their ablation by day 4, followed by secondary germ cell depletion by day 7.^9^ Accordingly, the partial loss of germ cells observed in the BC group likely reflects this secondary effect. In contrast, the Bus and combined Bus + BC (BBC) groups showed more extensive germ cell loss. Notably, the BBC group exhibited a trend toward even fewer residual germ cells compared to the Bus group (Figures 1G and 1H), suggesting that BC augmented germ cell ablation when used in combination. We then compared Sertoli cell ablation across treatment groups. The proportion of seminiferous tubules damaged by BC, as well as the number of Sertoli cells within these BC-damaged tubules, did not differ between the BC and BBC groups (Figures 1I and 1J). To evaluate potential off-target effects on other somatic cell populations, we quantified peritubular myoid cells and Leydig cells per tubule. No significant differences were observed across groups (Figures 1K and 1L), and vascular architecture remained intact with no evident abnormalities (Figure 1M).

### Host preconditioning and seminiferous tubule reconstruction in mouse allogeneic transplantation

**Figure 2:**
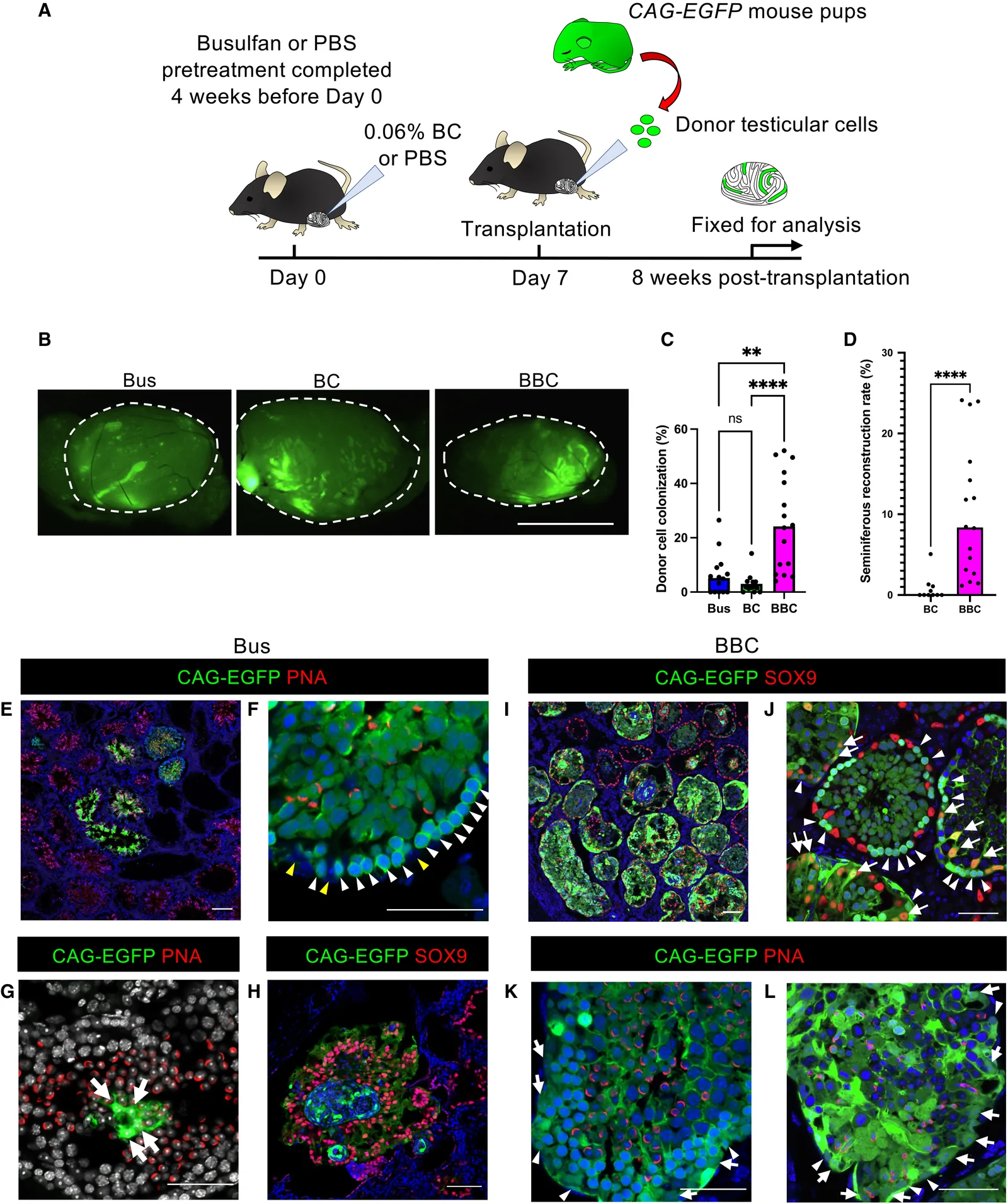
Host preconditioning enhances donor cell engraftment and seminiferous tubule reconstruction (A) Schematic overview of the allogeneic testicular cell transplantation experiment in mice preconditioned with busulfan (Bus), benzalkonium chloride (BC), and combined treatment (BBC). (B) Gross morphology of recipient testes at endpoint. Dashed lines indicate testicular contours. (C and D) Quantification of GFP^+^ donor cell engraftment and the proportion of seminiferous tubules reconstructed with both donor Sertoli cells and germ cells. Bars indicate the median, and each dot represents one analyzed testis. Five independent experiments were performed; *n* = 14, 13, and 16 analyzed testes for the Bus, BC, and BBC groups, respectively. (C) Quantification of GFP^+^ donor cell engraftment across groups. Statistical analysis was performed using the Kruskal-Wallis test followed by Dunn’s multiple-comparisons test; significant differences were observed between Bus and BBC groups (∗∗*p* = 0.0026) and between BC and BBC groups (∗∗∗∗*p* < 0.0001). (D) Proportion of seminiferous tubules reconstructed with both donor Sertoli cells and germ cells. Statistical analysis using the Mann-Whitney U test revealed a significant difference between the BC and BBC groups (∗∗∗∗*p* < 0.0001). Asterisks denote statistical significance as follows: ∗∗*p* < 0.01 and ∗∗∗∗*p* < 0.0001; ns, not significant. (E–H) Representative fluorescent images from Bus recipient testes showing donor-derived cells and their localization patterns. Tissues were immunostained for GFP (green). (E–G) were co-stained with PNA (red), and nuclei were counterstained with Hoechst (blue). (H) was co-stained with SOX9 (red), and nuclei were counterstained with Hoechst (gray). (E and F) Bus group recipient testes 62 days post-transplantation with 9.5 days postpartum (dpp) donor cells. (E) Donor-derived spermatogonia engrafted within recipient seminiferous tubules. (F) Engrafted donor spermatogonia were associated with host Sertoli cells, indicating support by the residual host niche. White arrowheads indicate donor-derived spermatogonia, and yellow arrowheads indicate host Sertoli cells. (G and H) Representative Bus group recipient testes 56 and 55 days post-transplantation, respectively, with 11.5 dpp donor cells. (G) Donor Sertoli cells ectopically localized within the seminiferous tubule lumen. Arrows indicate ectopically localized donor Sertoli cells. (H) Donor Sertoli cells accumulated in the rete testis. (I–L) Representative images from the BBC group showing co-engraftment of donor Sertoli and germ cells, with evidence of advanced spermatogenic differentiation indicated by PNA staining. Fluorescent images of recipient testes from the BBC group 56 days after transplantation with 6.5 dpp donor cells. Tissues were stained for GFP (green); SOX9 (red) in (I) and (J) and PNA (red) in (K) and (L). Nuclei were counterstained with Hoechst (gray). (I and J) Most seminiferous tubules containing donor cells exhibited co-engraftment of donor-derived Sertoli cells and spermatogonia. (I) Ectopic localization of Sertoli cells within the lumen of seminiferous tubules was also observed. (J) A representative image shows a tubule with donor spermatogonia alone (white arrowhead, center), and another tubule containing both donor Sertoli cells (white arrow) and spermatogonia (white arrowhead) co-localized within the same tubule. (K and L) Donor-derived spermatogenesis observed in reconstructed tubules with co-engrafted donor spermatogonia (white arrowheads) and Sertoli cells (white arrows). (K) PNA-positive round spermatids. (L) PNA-positive elongated spermatids. The representative images in (E–L) were selected from recipient testes analyzed across five independent transplantation experiments. Scale bars: 5 mm (B); 100 μm (E, H, and I); 50 μm (F, G, and J–L).

The aforementioned findings indicate that the BBC group exhibited a higher proportion of seminiferous tubules depleted of both germ cells and Sertoli cells compared to the BC group, suggesting that BBC treatment may serve as an effective host preconditioning strategy. To further evaluate its efficacy, we performed allogeneic transplantation using three different host groups: Bus, BC, and BBC (Figure 2A). Whole testicular cells isolated from juvenile CAG-EGFP mice were used as donor cells (Figure S1). Two months after transplantation, recipient testes were harvested and analyzed (Figure 2B). The number of testes showing engraftment of GFP-positive donor cells was 9 out of 14 (64%) in the Bus group, 10 out of 13 (77%) in the BC group, and 16 out of 16 (100%) in the BBC group. Moreover, the proportion of seminiferous tubules containing GFP-positive cells was highest in the BBC group, indicating enhanced donor cell colonization (Figure 2C). We next evaluated whether donor-derived germ cells and Sertoli cells co-localized within the same seminiferous tubules, a key criterion for structural reconstruction. Reconstructed seminiferous tubule-like structures were detected in 0 out of 14 testes (0%) in the Bus group, 4 out of 13 testes (31%) in the BC group, and all 16 of 16 testes (100%) in the BBC group. The proportion of reconstructed tubules per testis was significantly greater in the BBC group (median: 8.4%, range: 1.2%–24%) than in the BC group (median: 1.2%, range: 0.5%–5.1%) (Figure 2D). In the Bus group, most engrafted donor cells were spermatogonia, with spermatogenesis supported by residual host Sertoli cells (Figures 2E and 2F). Although donor-derived Sertoli cells were occasionally observed, they were rare and often localized within the tubule lumen (Figure 2G). In some cases, donor Sertoli cells formed aggregates in the rete testis, suggesting failed integration (Figure 2H). In contrast, the BBC group showed robust engraftment of both donor Sertoli cells and germ cells (Figure 2I). In some tubules, donor Sertoli cells were observed in the lumen alongside residual host Sertoli cells. Detailed analysis revealed that most seminiferous tubules in the BBC group contained both donor Sertoli cells and germ cells, while a minority contained donor spermatogonia alone (Figure 2J). To assess spermatogenic differentiation within reconstructed tubules, we examined donor-derived germ cell development in BBC-treated testes. Immunostaining with peanut agglutinin (PNA), a marker of acrosome development, revealed the presence of round and elongated spermatids, indicating advanced spermatogenic differentiation (Figures 2K and 2L). Taken together, these findings demonstrate that the BBC treatment regimen markedly improves the efficiency of seminiferous tubule reconstruction and donor-derived spermatogenesis compared to BC treatment alone.

### Xenogeneic seminiferous tubule reconstruction between rats and mice

**Figure 3:**
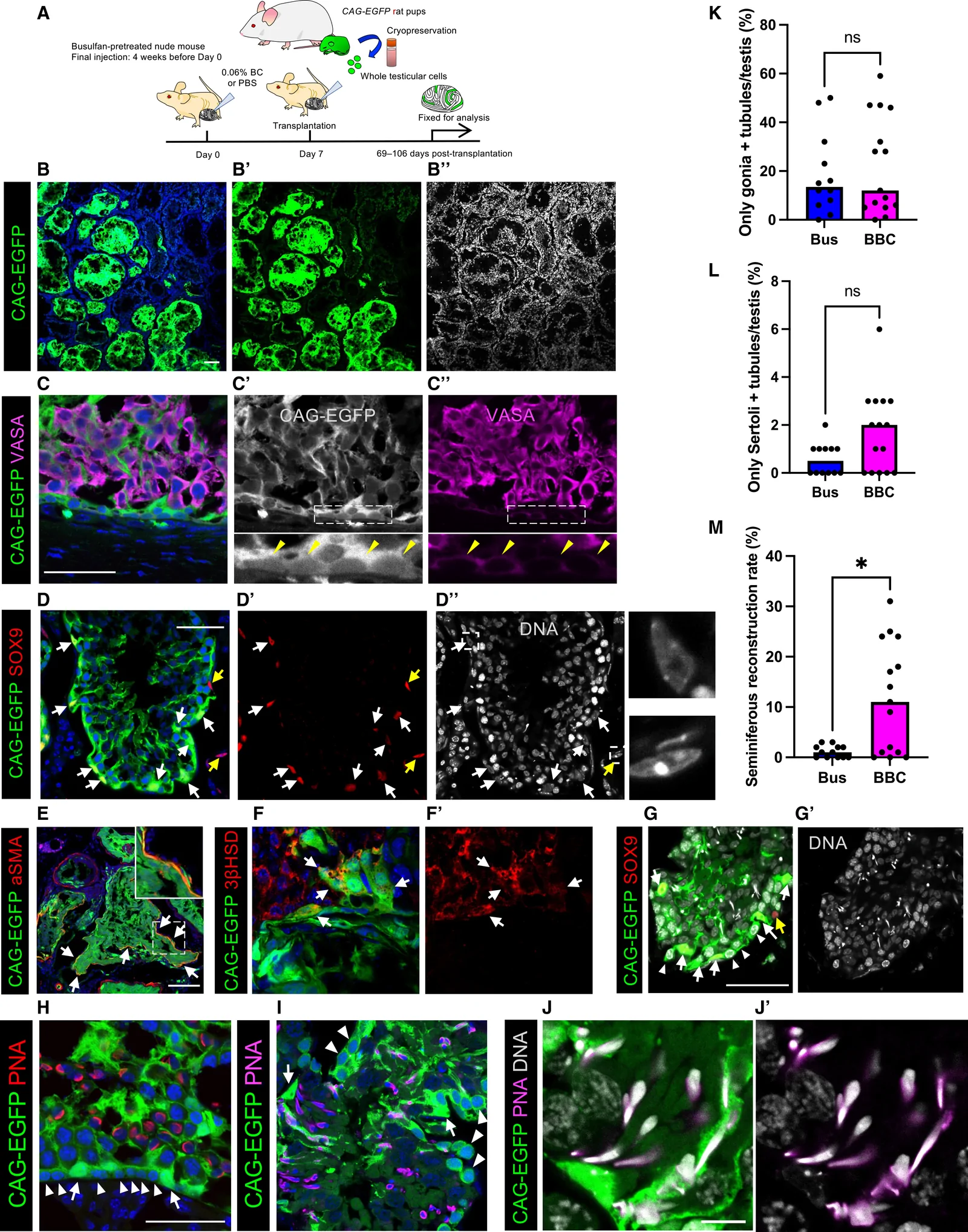
Xenogeneic reconstruction of seminiferous tubule-like structures with rat testicular cells in mouse hosts (A) Overview of transplantation protocol using cryopreserved rat cells and BBC-treated immunodeficient mice. (B–F’) BBC recipient testes at various time points after transplantation. CAG-EGFP (green) labels all transplanted cells. Nuclei are counterstained with Hoechst (blue or gray). (B–B”) Fluorescent images showing donor-derived rat cells colonizing mouse seminiferous tubules. BBC recipient testis 69 days post-transplantation. (C–C”) Donor VASA^+^ (magenta) spermatogonia colonized the basement membrane (arrowhead). (C’ and C”) The area enclosed by the dashed line is shown at higher magnification below. Yellow arrowheads indicate rat spermatogonia. (C’) shows GFP, and (C”) shows VASA only. (D–D′′) Donor SOX9^+^ (red) Sertoli cell colonization. Rat Sertoli cell (white arrows) and host mouse Sertoli cells (yellow arrowheads) were observed within the same seminiferous tubules 93 days post-transplantation. (D’) shows SOX9 staining only, and (D”) shows Hoechst (white) only. (D”) provides a view of the nuclear morphology of rat and host mouse Sertoli cells. A higher magnification view of each dashed region, showing a single Sertoli cell (rat or mouse, respectively), is presented on the right. Rat Sertoli cell is shown in the upper image, and mouse Sertoli cell in the lower image. (E–F’) Rat-derived peritubular myoid and Leydig cells detected in interstitial space. BBC recipient testis 106 days post-transplantation. (E) Rat PMCs (arrow; αSMA, red). The boxed area is enlarged on the right. (F) Rat Leydig cells expressing 3βHSD (red) colonized the interstitium (arrow). (F’) shows 3βHSD only. (G–J’) Evidence of spermatogenic progression, including PNA^+^ spermatids, within reconstructed tubules. (G and G’) Cryopreserved rat Sertoli cells (green, arrow), co-expressing SOX9 (red), successfully engrafted with rat spermatogonia (arrowheads) in the seminiferous tubules of BBC-treated host mouse testes. Host mouse Sertoli cells (yellow arrow). Hoechst (gray). (G’) Spermatogenic structures in rat seminiferous tubule-like structures. Hoechst (white) only. (H–J’) Rat spermatogenesis within reconstructed seminiferous tubules. PNA staining labeled round and elongated spermatids (red in H; magenta in I). Donor rat Sertoli cells (white arrows), donor rat spermatogonia (arrowheads). (H) BBC recipient testis 69 days post-transplantation. (I–J’) BBC recipient testis 106 days post-transplantation. (J and J’) Higher magnification images of a portion of the reconstructed seminiferous tubule-like structure shown in (I). (J) shows all channels, whereas (J’) is shown without the GFP channel to better visualize spermatid morphology. (K–M) Quantification of tubules containing donor-only spermatogonia, donor-only Sertoli cells, or both cell types. Bars indicate the median, and each dot represents one analyzed testis. Five independent experiments were performed. *n* = 12 and 15 analyzed testes for the Bus and BBC groups, respectively, for all analyses shown in (K–M). Statistical analysis was performed using the Mann-Whitney U test. (K) Percentage of tubules engrafted with only GFP^+^ rat spermatogonia in Bus and BBC groups. (L) Percentage of tubules containing only GFP^+^ rat Sertoli cells in Bus and BBC groups. (M) Quantification of the proportion of seminiferous tubules reconstructed with both donor-derived Sertoli cells and spermatogonia in Bus and BBC groups. The representative images in (B–J’) were selected from multiple BBC-treated recipient testes analyzed across five independent transplantation experiments (15 testes from 13 recipient mice).∗*p* = 0.0217. Asterisks denote statistical significance as follows: ∗*p* < 0.05, ns, not significant. Scale bars: 100 μm (B and E), 50 μm (C, D, and F–I), 10 μm (J).

Building upon our previous findings that the combination of Bus and BC (BBC) substantially enhances seminiferous tubule reconstruction, we next investigated whether this preconditioning strategy could be applied to xenogeneic transplantation. Specifically, we tested whether cryopreserved rat testicular cells could engraft and reconstruct seminiferous tubules within BBC-treated immunodeficient nude mice and included the Bus-treated group to allow comparison with previously reported rat spermatogenesis in mouse testes.^5^ To establish this xenogeneic model, nude mice were pretreated with three intraperitoneal injections of Bus on alternating days, followed by an intratubular injection of BC 4 weeks later to ablate endogenous germ and Sertoli cells. Seven days after BC treatment, cryopreserved testicular cells from CAG-EGFP transgenic immature rats were transplanted into the seminiferous tubules of recipient mice (Figures 3A and S2). Five independent transplantation experiments were conducted. In the Bus group, 6 mice (12 testes) received transplants. Transplantation was performed in 13 BBC-treated mice. Of the transplanted testes, 6 were excluded due to unsuccessful BC injection, and 5 were excluded due to technical failure, such as donor cell leakage into the interstitial space or insufficient cell delivery. The remaining 15 testes with successful BC injection and high-quality transplantation were included for analysis. Testes were harvested at a median of 86 days post-transplantation (range: 69–106 days). GFP^+^ donor rat cells were detected in every BBC-treated testis (15/15), colonizing both intra- and extra-tubular compartments (Figures 3B–3B”). Analysis revealed that donor rat spermatogonia (VASA-positive) and Sertoli cells (SOX9 positive) localized to the basement membrane of the seminiferous tubules, forming structurally organized, rat-derived seminiferous tubule-like structures within the mouse testis (Figures 3C–3D”). Rat-derived peritubular myoid cells (αSMA positive) and Leydig cells (3βHSD positive) were identified in the interstitial space, confirming successful engraftment of multiple somatic cell types (Figures 3E–3F’). A similar finding was also observed in our previously reported mouse-to-mouse syngeneic transplantation model.^9^ To determine whether rat spermatogenesis could proceed within the reconstructed tubules, histological and immunofluorescence analyses were performed. Nuclear-condensed cells resembling spermatids were observed in the reconstructed tubules of BBC-treated hosts (Figures 3G–3I). Furthermore, PNA staining confirmed the presence of GFP-positive round and elongating spermatids, indicating initiation and progression of rat spermatogenesis within the mouse testicular environment (Figures 3H–3J’). To assess the maturation status of spermatids, immunostaining for transition protein 1 (TNP1) was performed in combination with PNA.^10^ Cells positive for both PNA and TNP1 were detected, indicating that these spermatids correspond to elongating spermatids at steps 12–15, prior to the fully elongated stage (Figure S3). We also examined the pattern of donor cell integration. Tubules containing only donor spermatogonia were observed in all 15 BBC-treated testes (median occupancy: 12%, range: 0%–59%) and in 11 of 12 Bus testes (median: 14%, range: 0%–50%), with no significant difference in spermatogonia engraftment between the groups (Figures 3K and S4). However, seminiferous tubules containing only donor-derived Sertoli cells were more prevalent in the BBC group. In this group, 10 of 15 testes exhibited such tubules (median occupancy: 2%, range: 0%–6%), whereas only 6 of 12 testes in the Bus group exhibited them (median: 0.5%, range: 0%–2%) (Figure 3L). To quantify reconstruction efficiency, immunohistochemical analysis was performed. Among the 15 BBC-treated testes, 12 (80%) exhibited seminiferous tubule reconstruction, with a median of 11% (range: 0%–31%) of tubules containing rat-derived structures (Figure 3M). In contrast, in the Bus group, 7 of 12 testes showed reconstructed tubules, but with a significantly lower median occupancy of 1% (range: 0%–3%) (Figures 3M and S4). Collectively, these findings demonstrate that BBC-based host preconditioning facilitates efficient xenogeneic seminiferous tubule reconstruction and supports rat spermatogenic differentiation in the mouse testis—an important step toward cross-species testicular niche engineering.

## Discussion

This study demonstrates the feasibility of reconstructing xenogeneic seminiferous tubule-like structures by employing a host preconditioning strategy that combines Bus-mediated germ cell ablation with BC-induced Sertoli cell ablation. Compared to Bus or BC treatment alone, the combined BBC approach significantly enhanced the efficiency of donor cell engraftment and subsequent spermatogenic differentiation within reconstructed seminiferous tubule-like structures.

Initially, we hypothesized that Bus-induced germ cell ablation would reduce testicular volume, thereby allowing a greater volume of BC solution to infiltrate the seminiferous tubules and enhance Sertoli cell ablation. However, neither the proportion of Sertoli cell-depleted tubules nor the number of residual Sertoli cells in BC-damaged tubules differed significantly between the BC and BBC groups. Despite this, the improved efficiency of donor cell engraftment observed in the BBC group may be attributed to the prior elimination of stratified germ cells by Bus. This pre-removal likely minimized secondary germ cell death caused by BC-mediated Sertoli cell ablation, thereby reducing acute testicular inflammation and creating a more permissive microenvironment for donor cell colonization. In addition, the absence of germ cells may have increased the available luminal space within the seminiferous tubules, enabling the injection of a larger volume of donor cells. Together, these factors may account for the enhanced donor cell engraftment and seminiferous tubule reconstruction observed following BBC preconditioning. Moving forward, further studies are warranted to dissect the molecular and cellular mechanisms underlying this improvement and to evaluate the generalizability of this strategy across different xenogeneic transplantation models.

To improve donor cell engraftment efficiency, the use of severely immunodeficient mouse strains may be advantageous over conventional nude (nu) mice. NOG mice, which are now commercially available from various suppliers, represent a promising platform for xenogeneic transplantation studies, particularly in large mammals and potentially even in human reproductive biology.^11^ Conversely, more rigorous ablation of endogenous spermatogonia may also enhance the success of xenogeneic spermatogenesis. However, the use of additional alkylating agents in immunodeficient hosts raises safety concerns. Alternative approaches, such as localized abdominal irradiation, may provide a viable solution.^12^ In parallel, pharmacological strategies aimed at preserving donor-derived spermatogonia following transplantation represent another promising avenue to improve engraftment outcomes.^13^

Sertoli cells are known to possess immunomodulatory properties, enabling them to promote immune tolerance in transplantation contexts.^14^ Previous studies have demonstrated successful Sertoli cell transplantation across different mouse strains, highlighting their immune-privileged nature.^15^ Moreover, co-transplantation of Sertoli cells with pancreatic islet cells has been shown to improve engraftment and survival.^14^ This immunoprotective capacity is thought to support the engraftment of other testicular cell types, including spermatogonia, Leydig cells, and peritubular myoid cells. Given that spermatogenesis is orchestrated not only by systemic hormonal cues but also by intricate local cell-cell interactions,^8^^,^^16^^,^^17^ the strategic transplantation of heterologous cell populations into their appropriate niches may further enhance cross-species spermatogenic differentiation within engineered niches.

The anatomical structure and spatial positioning of the testes vary across species.^18^^,^^19^^,^^20^ Testicular temperature is a well-established determinant of spermatogenesis.^21^ The induction of rat spermatogenic differentiation within the mouse testis—despite these interspecies differences—suggests that xenogeneic transplantation models may be useful for future studies of cross-species testicular niche engineering.

Xenogeneic spermatogonial transplantation may inform future fertility preservation strategies, particularly in prepubertal cancer patients undergoing gonadotoxic therapies. If future studies establish further sperm maturation, fertilization competence, and safety, xenogeneic transplantation approaches may inform strategies for individuals facing infertility. Moreover, xenogeneic transplantation provides a key advantage by minimizing the risk of malignant cell contamination that can occur during autologous transplantation,^22^ thereby providing a rationale for further investigation in fertility preservation models. Beyond biomedical research, this approach also holds significant potential for species conservation by enabling the propagation of endangered animals through spermatogonial transplantation. Despite these promising findings, several challenges remain. Although we successfully demonstrated the reconstruction of xenogeneic seminiferous tubule-like structures and spermatogenesis, we cannot completely exclude the possibility that rat spermatogenesis within the reconstructed tubules was partially supported by residual host mouse Sertoli cells. Further validation using donor testicular cells from species whose spermatogenesis is not supported by mouse Sertoli cells, such as primates, will be required to more definitively establish cross-species spermatogenic differentiation.

For future clinical translation, it will be essential to perform functional assessments of sperm derived from reconstructed seminiferous tubules using techniques such as intracytoplasmic sperm injection (ICSI). In addition, transcriptomic and epigenetic analyses should be conducted to identify any aberrant gene expression profiles or epigenetic alterations. Further studies would be needed to consider whether host-derived microbes are also transferred.^23^ Finally, even if fertilization-competent sperm can be generated, ethical considerations must be systematically addressed before advancing toward broader applications.^24^^,^^25^

This study establishes a robust and versatile platform for xenogeneic spermatogonial transplantation by demonstrating that simultaneous engineering of the SSC niche using donor cells can significantly improve donor cell engraftment, seminiferous tubule-like structure reconstruction, and cross-species spermatogenic differentiation. Importantly, our findings extend beyond previous xenogeneic transplantation studies by demonstrating the reconstruction of seminiferous tubule-like structures, rather than relying on pre-existing host architecture. These findings may inform future studies in reproductive biology, fertility preservation, and cross-species testicular niche engineering.

### Limitations of the study

This study has several limitations. First, although advanced spermatogenic differentiation was observed, the functional competence of the generated spermatozoa was not directly evaluated. Specifically, functional assays such as ICSI or *in vitro* fertilization (IVF) were not performed, and therefore, the fertilization capacity of these cells remains to be determined. In addition, this study did not establish epididymal transport, the presence of donor-derived epididymal sperm, or endocrine function of the reconstructed seminiferous tubule-like structures. Second, we cannot completely exclude the possibility that residual host Sertoli cells contributed to the observed spermatogenesis within the reconstructed seminiferous tubule-like structures. Future studies incorporating xenogeneic transplantation using donor cells from species whose spermatogenesis is not supported by mouse Sertoli cells, such as primates, together with functional, epididymal, and endocrine assessments, will enable more rigorous validation of donor-derived spermatogenic differentiation and further clarify the potential of this approach.

## Resource availability

### Lead contact

Further information and requests for resources and reagents should be directed to and will be fulfilled by the lead contact, Tetsuhiro Yokonishi (yokonishi@med.kawasaki-m.ac.jp).

### Materials availability

This study did not generate new unique reagents. Materials are available from the corresponding author upon reasonable request.

### Data and code availability

• All data reported in this paper are available within the paper and supplemental information. Microscopy data reported in this paper will be shared by the lead contact upon request.
• This paper does not report original code.
• Any additional information required to reanalyze the data reported in this paper is available from the lead contact upon request.

## Acknowledgments

We thank Iordan Stefanov Batchvarov, Kimiko Hagihara, and Mari Inada for their support in mouse colony maintenance and technical assistance. This work was supported by 10.13039/501100001700MEXT/10.13039/501100001691JSPS 10.13039/501100001691KAKENHI (grant nos. JP20K22748 and 23K21472 to T.Y.). Additional financial support was provided by the Teraoka Scholarship Foundation, 10.13039/501100008672Yamaguchi Endocrine Research Foundation, Okayama Medical Foundation, Terumo Life Science Foundation, and Research Project Grant R02B-011 from 10.13039/501100009158Kawasaki Medical School.

## Author contributions

T.Y. generated hypotheses and designed and carried out the experiments. B.C. supervised all experiments. T.Y. and B.C. wrote the paper. All authors reviewed/commented on this work/paper.

## Declaration of interests

The authors are inventors on a patent related to the niche ablation technology used in this study (WO/2019/108973; PCT/US2018/063374). The methods described here represent an improved application of this patented technology.

## Declaration of generative AI and AI-assisted technologies in the writing process

During the preparation of this work, the authors used ChatGPT exclusively in order to improve the grammar, spelling, syntax, clarity, and fluency of the English text. After using this tool/service, the authors reviewed and edited the content as needed and take full responsibility for the content of the publication.

## STAR★Methods

### Key resources table

**Table:** 

REAGENT or RESOURCE	SOURCE	IDENTIFIER
**Experimental models: organisms/strains**		

Mouse: C57BL/6J	The Jackson Laboratory	Strain #000664; RRID: IMSR_JAX:000664
Mouse: C57BL/6JmsSlc	Japan SLC, Inc.	C57BL/6JmsSlc
Mouse: C57BL/6-Tg(CAG-EGFP)	Japan SLC, Inc.	C57BL/6-Tg(CAG-EGFP)C14-Y01-FM131Osb; RBRC00267
Rat: SD-Tg(CAG-EGFP)	Japan SLC, Inc.	SD-Tg(CAG-EGFP)4Osb; NBRP Rat No. 0235
Mouse: BALB/cSlc-nu/nu	Japan SLC, Inc.	BALB/cSlc-nu/nu; Foxn1nu

**Chemicals, peptides, and recombinant proteins**		

PNA lectin (AF568)	Thermo Fisher Scientific (Invitrogen)	Cat# L32458
PNA lectin (AF647)	Thermo Fisher Scientific (Invitrogen)	Cat# L32460
Benzalkonium chloride (BC)	ACROS Organics	Cat# 21541–0010; Lot# A0422482
Phosphate-buffered saline (PBS)	FUJIFILM Wako Pure Chemical Corporation	Cat# 162–19321; Lot# WTL5636
Busulfan	Sigma-Aldrich	Cat# B2635-25G
N,N-Dimethylacetamide (DMA)	FUJIFILM Wako Pure Chemical Corporation	Cat# 042-02546
Polyethylene glycol 400 (PEG 400)	FUJIFILM Wako Pure Chemical Corporation	Cat# 161-09065
DMEM	Gibco	Cat# 11995-065
Fetal bovine serum (FBS)	Gibco	Cat# 10438-026
Albumin, from Bovine Serum, Fraction V, pH 7.0	FUJIFILM Wako Pure Chemical Corporation	Cat# 011–27055; Lot# SKJ2849
Penicillin-Streptomycin Solution (×100)	FUJIFILM Wako Pure Chemical Corporation	Cat# 168–23191; Lot# TPJ7020
Trypsin	Gibco	Cat# 15090-046
EDTA	Nacalai Tesque, Inc.	Cat# 15111-32
CELLBANKER 1	NIPPON ZENYAKU KOGYO Co., Ltd.	Cat# CB011
Bromophenol blue	Sigma-Aldrich	Cat# 114391; Lot# BCCD6533
Trypan Blue Stain (0.4%)	Gibco	Cat# 15250–061; Lot# 2283795
Tissue-Tek O.C.T. Compound	Sakura Finetek Japan	Cat# 45833; Lot# 4675-00
32% Paraformaldehyde Solution	MUTO PURE CHEMICALS Co., Ltd.	Cat# 17711; Lot# 220418
Triton X-100	Nacalai Tesque, Inc.	Cat# 35501–15; Lot# M0T7195
Mounting medium (polyvinyl alcohol/glycerol/0.2M Tris-HCl, pH 8.0)	Prepared in-house	N/A
Polyvinyl alcohol	MP Biomedicals	Cat# 151937; Lot# S1581
Glycerol	Nacalai Tesque, Inc.	Cat# 17018–25; Lot# V5K7386
1 M Tris-HCl (pH 8.0)	Nippon Gene Co., Ltd.	Cat# 312-90061
2,2,2-Tribromoethanol	Sigma-Aldrich	Cat# T48402
2-Methyl-2-butanol (tert-amyl alcohol)	Sigma-Aldrich	Cat# 152463
Midazolam Injection 10 mg “NIG”	Nichi-Iko Pharmaceutical Co., Ltd.	Lot# HC0011
Vetorphale 5 mg	Meiji Animal Health Co., Ltd.	Lot# VETLI5
Domitor 10 mL	NIPPON ZENYAKU KOGYO Co., Ltd.	Lot# 411140
Physiological saline	Otsuka Pharmaceutical Factory, Inc.	Lot# K5E96

**Antibodies**		

Chicken anti-GFP antibody	Aves	Cat# GFP-1020; RRID: AB_10000240
Rabbit anti-SOX9 antibody	Millipore	Cat# AB5535; RRID: AB_2239761
Cy3 anti-αSMA	Sigma-Aldrich	Cat# C6198; Clone 1A4; RRID: AB_476856
Rat anti-PECAM1	BD Biosciences	Cat# 557355; Clone MEC13.3; RRID: AB_396660
Rabbit anti-VASA/DDX4	Abcam	Cat# ab13840; RRID: AB_443012
Rat anti-TRA98	Abcam	Cat# ab82527; Clone TRA98; RRID: AB_1659152
Rabbit anti-3βHSD	TransGenic Inc.	Cat# KO607; RRID: AB_2722746
Rabbit anti-Laminin	Gift from Harold Erickson (Duke University)	N/A
Rabbit anti-TNP1	Proteintech	Cat# 17178-1-AP; RRID: AB_2206757
AF488 Donkey anti-Chicken	Jackson ImmunoResearch	Cat# 703-545-155; RRID: AB_2340375
AF555 goat anti-rabbit	Life Technologies	Cat# A-21429; RRID: AB_2535850
AF647 goat anti-rabbit	Life Technologies	Cat# A-21244; RRID: AB_2535812
AF555 goat anti-rat	Life Technologies	Cat# A-21434; RRID: AB_2535855

**Other**		

Nalgene General Long-Term Storage Cryogenic Tubes	Thermo Scientific	Cat# 5000–0020; Lot# 1307020
Cellstain® Hoechst 33342 solution	Dojindo Laboratories	Cat# H342
BICELL	Nihon Freezer Co., Ltd.	N/A
Cell Strainer 40 μm Nylon	AS ONE	Cat# 3-6649-01
GFP detection LED handheld light	OptoCode Co., Ltd.	Model# LEDGFP-3WOF

**Software**		

Fiji software	NIH	v1.54f
GraphPad Prism	GraphPad	v9

### Experimental model and study participant details

#### Animals

C57BL/6J inbred mice (Mus musculus) were obtained from The Jackson Laboratory (Bar Harbor, ME, USA) and C57BL/6JJmsSlc mice, BALB/cSlc-nu/nu immunodeficient mice (Mus musculus), C57BL/6-Tg(CAG-EGFP)^26^ transgenic mice (Mus musculus), and SD-Tg(CAG-EGFP)^26^ transgenic rats (Rattus norvegicus) were obtained from Japan SLC (Shizuoka, Japan). For transplantation experiments, donor testes were harvested from male CAG-EGFP mouse or rat pups at 4.5–11.5 days postpartum (dpp), and transplanted into adult male C57BL/6JmsSlc or BALB/cSlc-nu/nu host mice aged 8–12 weeks. Animals were housed in individually ventilated cages under specific pathogen-free conditions. At Duke University, mice were maintained under a 12 h light/12 h dark cycle at 22 ± 0.1°C with 30–70% relative humidity. At Kawasaki Medical School, mice and rats were maintained in standard laboratory cages on a 12 h light/12 h dark cycle at a constant temperature of 24 ± 1 °C. Food and water were provided *ad libitum* in both facilities. Donor pups were euthanized before testis collection by decapitation under approved institutional procedures. Recipient animals were euthanized at the experimental endpoint by CO_2_ inhalation followed by cervical dislocation. All animal procedures were conducted in accordance with the guidelines of the National Institutes of Health and all relevant institutional and national regulations for the care and use of laboratory animals. Experimental protocols were approved by the Institutional Animal Care and Use Committee of Duke University Medical Center (protocol no. A126-17-05) and the Institutional Committee for Laboratory Animal Experimentation of Kawasaki Medical School (protocol no. 22–041, 24–062, 25–125).

### Method details

#### BC solution preparation

A commercially available 20% (w/v) benzalkonium chloride (BC) solution (ACROS Organics, Cat# 21541–0010, lot no. A0422482) was diluted in phosphate-buffered saline (PBS; FUJIFILM Wako Pure Chemical Co., Cat# 162–19321, lot no. WTL5636) to prepare working solutions at final concentrations ranging from 0.02% to 0.06%.^27^

#### Busulfan solution preparation

Busulfan solution was prepared by dissolving 8.75 mg of busulfan (Sigma-Aldrich, Cat# B2635-25G) in 2 mL of N,N-dimethylacetamide (DMA). After complete dissolution, 4 mL of polyethylene glycol 400 (PEG 400) and 4 mL of water were added sequentially and mixed thoroughly. The final solution contained 0.875 mg/mL busulfan in DMA/PEG 400/water at a volume ratio of 20:40:40. The solution was administered intraperitoneally at 10 mL/kg, corresponding to a busulfan dose of 8.75 mg/kg per injection. Accordingly, a 20-g mouse received 0.2 mL of the solution, containing 0.175 mg of busulfan.

#### Recipient preconditioning and host preparation

For the BC dose-optimization experiments, recipient testes received rete testis injections of PBS or BC at final concentrations of 0.02%, 0.04%, or 0.06% without prior busulfan treatment. For transplantation experiments, the day of rete testis injection of BC or PBS was designated as day 0. Recipient mice were assigned to the Bus, BC, or BBC group. Bus recipients received three intraperitoneal injections of the prepared busulfan solution at 8.75 mg/kg per injection on days −32, −30, and −28, as previously described,^28^ followed by a rete testis injection of PBS on day 0 as a control treatment. BC recipients received three intraperitoneal injections of PBS at the same volume as the busulfan injections on days −32, −30, and −28, followed by a rete testis injection of 0.06% BC in PBS on day 0. BBC recipients received the same busulfan regimen as the Bus group, followed by a rete testis injection of 0.06% BC in PBS on day 0. Donor testicular cells were transplanted on day 7, seven days after the BC or PBS injection. For xenogeneic transplantation experiments, only the Bus and BBC groups were used.

Host mice were anesthetized via intraperitoneal injection of either 1.25% Avertin (2,2,2-tribromoethanol; 250 mg/kg body weight)^28^ or a combination of medetomidine, midazolam, and butorphanol (0.3, 4.0, and 5.0 mg/kg body weight, respectively). Animals were monitored until full recovery from anesthesia and were subsequently checked at least once daily for 3 days for general condition, body weight, wound healing, pain-related behavior, mobility, and signs of distress. Humane endpoints included marked body weight loss, persistent lethargy or immobility, wound dehiscence or infection, impaired access to food or water, or persistent pain or distress unresponsive to supportive care or analgesic treatment. BC solution (18 μL) was mixed with 2 μL of 0.2% bromophenol blue (Sigma-Aldrich, Cat# 114391) to visualize the injection. The resulting solution was injected into the rete testis using a glass capillary needle. Approximately 10–15 μL was delivered per testis, with injection continued until the seminiferous tubules were visibly filled with the blue solution.^29^ Successful injection was defined as visible filling of the seminiferous tubules without substantial leakage into the interstitial space. For the Bus group, PBS alone was administered using the same rete testis injection procedure.

#### Donor mouse testicular cell preparation and transplantation

On the day of transplantation, testes were dissected from male 5.5–11.5 dpp CAG-EGFP mouse pups. Donor pups used for testicular cell preparation were euthanized as described above before tissue collection. Testicular cells were enzymatically dissociated in 0.25% trypsin and 1 mM EDTA at 37 °C for 10 min. The reaction was terminated by adding serum, and the cells were washed twice with Dulbecco’s Modified Eagle Medium (DMEM; Gibco, Cat# 11995–065) supplemented with 10% fetal bovine serum (FBS; Gibco, Cat# 10438–026) and penicillin-streptomycin (P/S). The cell suspension was filtered through a 40 μm nylon mesh and resuspended in DMEM containing 10% FBS and P/S.^30^ Cell viability was assessed using trypan blue exclusion (Gibco, Cat# 15250–061), and found to be high (85–100%). The cell suspension was further diluted in the same medium to obtain a final concentration of 1.2 × 10^8^ cells/mL for transplantation. An 18-μL aliquot of the cell suspension was mixed with 2 μL of 0.4% trypan blue, yielding a final trypan blue concentration of 0.04%. Approximately 10–15 μL of the resulting suspension was injected into the seminiferous tubules of host mice via the rete testis 7 days after BC or PBS injection. Injections were performed using a glass capillary needle back-loaded with the cell suspension and the distribution of trypan blue was used to visualize injection efficiency. All rete testis injections and transplantation procedures were performed under general anesthesia as described above, with perioperative analgesia and postoperative monitoring conducted in accordance with the approved animal protocol.

#### Donor rat testicular cell cryopreservation and transplantation

CAG-EGFP rats were identified under blue LED illumination (LEDGFP-3WOF; excitation 470 ± 20 nm, emission >540 nm; OptoCode, Tokyo, Japan). Testes were collected from male 4.5–7.5 dpp CAG-EGFP rat pups, and testicular cells were isolated using the same enzymatic procedure described above for mouse testicular cells, collected by centrifugation, and resuspended at a final density of 5 × 10^5^ cells/mL. Donor rat pups were euthanized as described above before testis collection. Cells were suspended in CELLBANKER 1 cryopreservation medium (NIPPON ZENYAKU KOGYO Co., Ltd., Cat# CB011) and dispensed into 2.0 mL cryovials (Thermo Scientific, Cat# 5000–0020). Samples were placed in a controlled-rate freezing container (BICELL, Nihon Freezer Co., Ltd.) and cooled overnight at −80 °C. The following morning, cryovials were transferred to liquid nitrogen for long-term storage. The mean cryopreservation period was 56 days (range: 33–78 days). Prior to use, samples were rapidly thawed in a 37 °C water bath. After thawing, cells were diluted with 1 mL of DMEM containing 10% FBS and P/S, centrifuged at 300 × g for 5 min, and resuspended in fresh medium. Cell viability after thawing was 80–95%, and the final cell concentration used for transplantation was adjusted to 1.2 × 10^8^ cells/mL. An 18-μL aliquot of the cell suspension was mixed with 2 μL of 0.4% trypan blue, yielding a final trypan blue concentration of 0.04%. Rat testicular cell transplantation was performed using the same rete testis injection procedure described above for mice. Approximately 10–15 μL of the cell suspension was injected into each recipient testis via the rete testis 7 days after BC treatment.

#### Evaluation of seminiferous tubule reconstruction

Seminiferous tubule reconstruction was evaluated using stereomicroscopic observation and histological analysis. The development of donor-derived CAG-EGFP-positive tubules and the progression of spermatogenesis were assessed by detecting GFP fluorescence with a stereomicroscope equipped with a GFP excitation light source (DP70; Olympus). For histological analysis, testes were fixed in Bouin’s solution, embedded in paraffin, and sectioned at 3 μm thickness. A single section representing the largest cross-sectional area was obtained from each specimen and stained with hematoxylin and eosin (H&E). A reconstructed xenogeneic seminiferous tubule-like structure was defined as a tubule containing GFP-positive donor-derived Sertoli cells together with donor-derived germ cells. Spermatogenesis was considered present when differentiated germ cells, including PNA-positive spermatids, were detected within the tubule.

#### Immunofluorescence and image analysis

Host testes were fixed in 4% paraformaldehyde in PBS at 4 °C overnight, followed by cryoprotection in a sucrose gradient (5%, 10%, 15%, 20%, and 50% in PBS). Samples were incubated overnight at 4 °C in a 1:1 mixture of 50% sucrose and OCT compound (Sakura Finetek), then embedded in OCT.^31^ Cryosections (8–12 μm) were cut and incubated in blocking solution (PBTx, 10% FBS, 3% BSA) for 30 min. PBTx consisted of PBS containing 0.2% Triton X-100. Antigen retrieval was not performed. Primary antibodies diluted in blocking solution were applied overnight at 4 °C. The primary antibodies used were chicken anti-GFP antibody (1:2,000), rabbit anti-SOX9 antibody (1:2,000), Cy3-conjugated anti-αSMA antibody (1:2,000), rat anti-PECAM1 antibody (1:250), rabbit anti-VASA/DDX4 antibody (1:1,000), rat anti-TRA98 antibody (1:1,000), rabbit anti-3βHSD antibody (1:250), rabbit anti-laminin antibody (1:1,000), and rabbit anti-TNP1 antibody (1:500). Alexa Fluor 568-conjugated PNA lectin and Alexa Fluor 647-conjugated PNA lectin were each reconstituted in 1 mL of distilled water and used at a dilution of 1:5,000. Following primary antibody incubation, sections were washed three times for 5 min each with PBTx. Secondary antibodies were incubated for 1 h at room temperature in the dark. The secondary antibodies used were Alexa Fluor 488 donkey anti-chicken IgG, Alexa Fluor 555 goat anti-rabbit IgG, Alexa Fluor 647 goat anti-rabbit IgG, and Alexa Fluor 555 goat anti-rat IgG, each diluted 1:500 in PBTx. After secondary antibody incubation, sections were washed three times for 5 min each with PBS. Nuclei were counterstained with Hoechst 33342, and samples were mounted in a Mowiol-based mounting medium prepared with 4.8 g Mowiol (polyvinyl alcohol; MP Biomedicals, Cat# 151937), 12 g glycerol, 12 mL water, and 24 mL of 0.2 M Tris-HCl (pH 8.0). Confocal images were acquired as single optical sections; z stack imaging was not performed. Brightness and contrast were adjusted identically across images within each comparison. Imaging was performed using Zeiss LSM 700 or 780 confocal microscopes. Immunofluorescence images were analyzed using Fiji (v1.54f), and statistical analysis was conducted with GraphPad Prism (v9).

#### Testicular cell immunostaining

Testicular cells were suspended in solution and dropped onto glass slides, followed by a 30-min incubation. After removing the supernatant, cells were fixed with 4% paraformaldehyde at room temperature for 30 min. Samples were washed with PBS and permeabilized with PBTx for 5 min, twice. Blocking was performed using the same blocking solution as described above for 30 min at room temperature. Cells were then incubated overnight at 4 °C with primary antibodies diluted in blocking solution. After three washes with PBTx, samples were incubated with fluorophore-conjugated secondary antibodies for 1 h at room temperature in the dark. After three washes with PBS, nuclei were counterstained with Hoechst 33342, and the slides were mounted using the polyvinyl alcohol-based mounting medium described above.

### Quantification and statistical analysis

Fiji (v1.54f) was used to quantify the proportion of seminiferous tubules affected by BC. This was calculated by counting αSMA-defined seminiferous tubule cross-sections in 3–5 sections per testis and identifying affected tubules as those lacking SOX9^+^ Sertoli cells within the tubule cross-section. The numbers of Sertoli cells, germ cells (TRA98^+^ or VASA^+^), Leydig cells (3βHSD^+^), and peritubular myoid cells (PMCs; αSMA^+^) were also quantified. Cell numbers were counted in defined tubule cross-sections or in the interstitial areas surrounding each tubule, as appropriate for each cell type. Leydig cells and PMCs were counted in the interstitial areas surrounding each tubule. Donor cell engraftment and tubule reconstruction were quantified in seminiferous tubule cross-sections based on GFP expression and cell-type marker staining. Tubules were classified as containing GFP^+^ donor-derived germ cells only, GFP^+^ donor-derived Sertoli cells only, or both cell types, and each category was expressed as a proportion of all analyzed seminiferous tubule cross-sections per testis. For all quantifications, the experimental unit and definition of n are specified in the corresponding figure legends. Depending on the analysis, each data point represents an individual recipient mouse, an individual analyzed testis, or the median value obtained from three mice within one independent experiment. For the transplantation experiments shown in Figures 2 and 3, each analyzed testis was quantified separately, and n represents the number of analyzed testes. The numbers of analyzed mice, testes, and independent experimental replicates are provided in the corresponding figure legends. Before quantitative analysis, testes subjected to BC injection were excluded if BC delivery was unsuccessful. Testes were also excluded if transplantation was technically unsuccessful because of substantial donor-cell leakage into the interstitial space or insufficient cell delivery into the seminiferous tubules. Poorly preserved sections, damaged tissue areas, and regions with inadequate immunostaining were excluded from quantification. Data are presented as medians. Statistical analyses were performed using GraphPad Prism version 9. Comparisons among three or more groups were performed using the Kruskal–Wallis test followed by Dunn’s multiple-comparisons test, whereas comparisons between two groups were performed using the Mann–Whitney U test. Statistical significance was defined as *p* < 0.05. Exact *p* values, the statistical test used, and the definition of n for each analysis are provided in the corresponding figure legends.
