# Supplementary material for: Reconstruction of xenogeneic seminiferous tubule-like structures in mouse testis using cryopreserved rat testicular cells
Source: iScience. 2026 Aug 13;29(9):117223. doi: 10.1016/j.isci.2026.117223 (PMC13495095; doi:10.1016/j.isci.2026.117223)
Supplement: Supplementary file 1 [file mmc1.pdf]

iScience, Volume *29*

Supplemental information

Reconstruction of xenogeneic seminiferous tubule-like structures in mouse testis using cryopreserved rat testicular cells

Tetsuhiro Yokonishi and Blanche Capel

Figure S1. Characterization of donor mouse testicular cells derived from CAG-EGFP mouse pups.

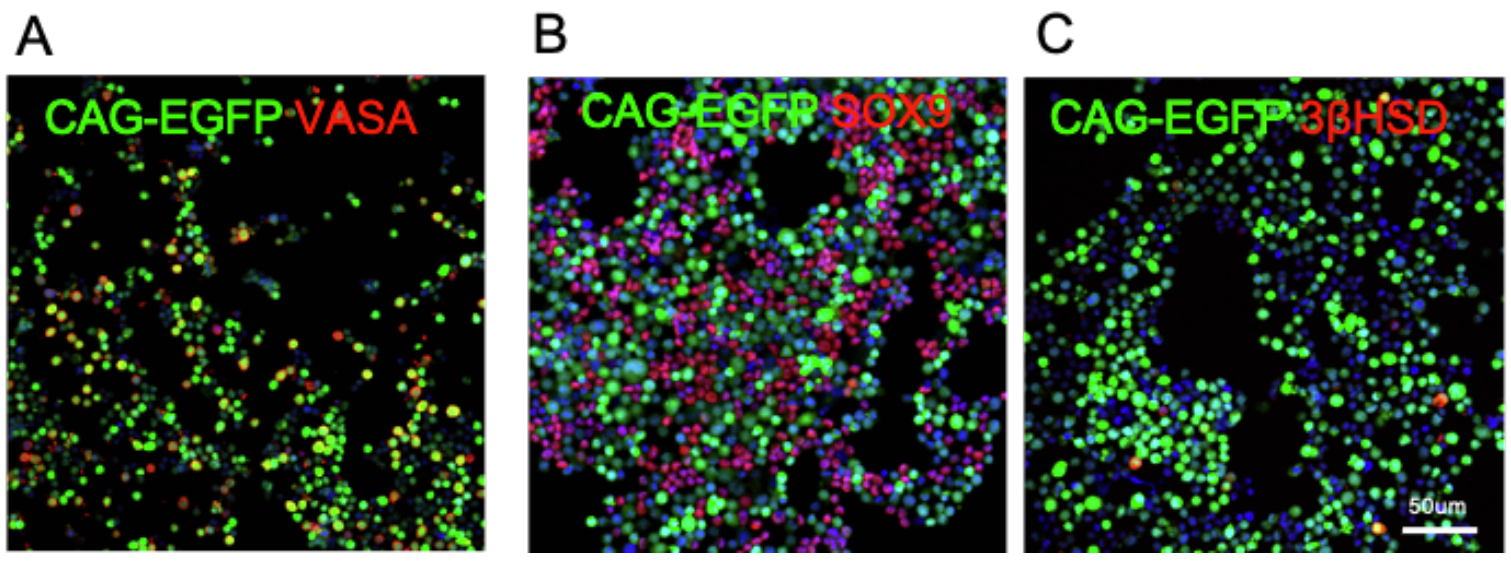

D

Mouse donor testicular cells (n=4)

Cell type	Population (%)
Spermatogonia	44.8 (30-60)
Sertoli cell	23.0 (11-42.6)
Leydig cell	4.01 (1.6-13)

(A–C) Immunohistochemical analysis of 5.5 dpp donor cells without anti-GFP antibody staining. Nuclei were counterstained with Hoechst (blue). (A) Donor spermatogonia were positive for VASA (red). (B) Donor Sertoli cells were labeled with anti-SOX9 antibody (red). (C) Donor Leydig cells were identified by anti-3βHSD antibody (red). (D) Cell population analysis of donor testicular cells. Images in (A–C) are representative of four biologically independent experiments. Data in (D) were obtained from four biologically independent experiments.

Figure S2. Characterization of donor testicular cells and testes from CAG-EGFP rat pups.

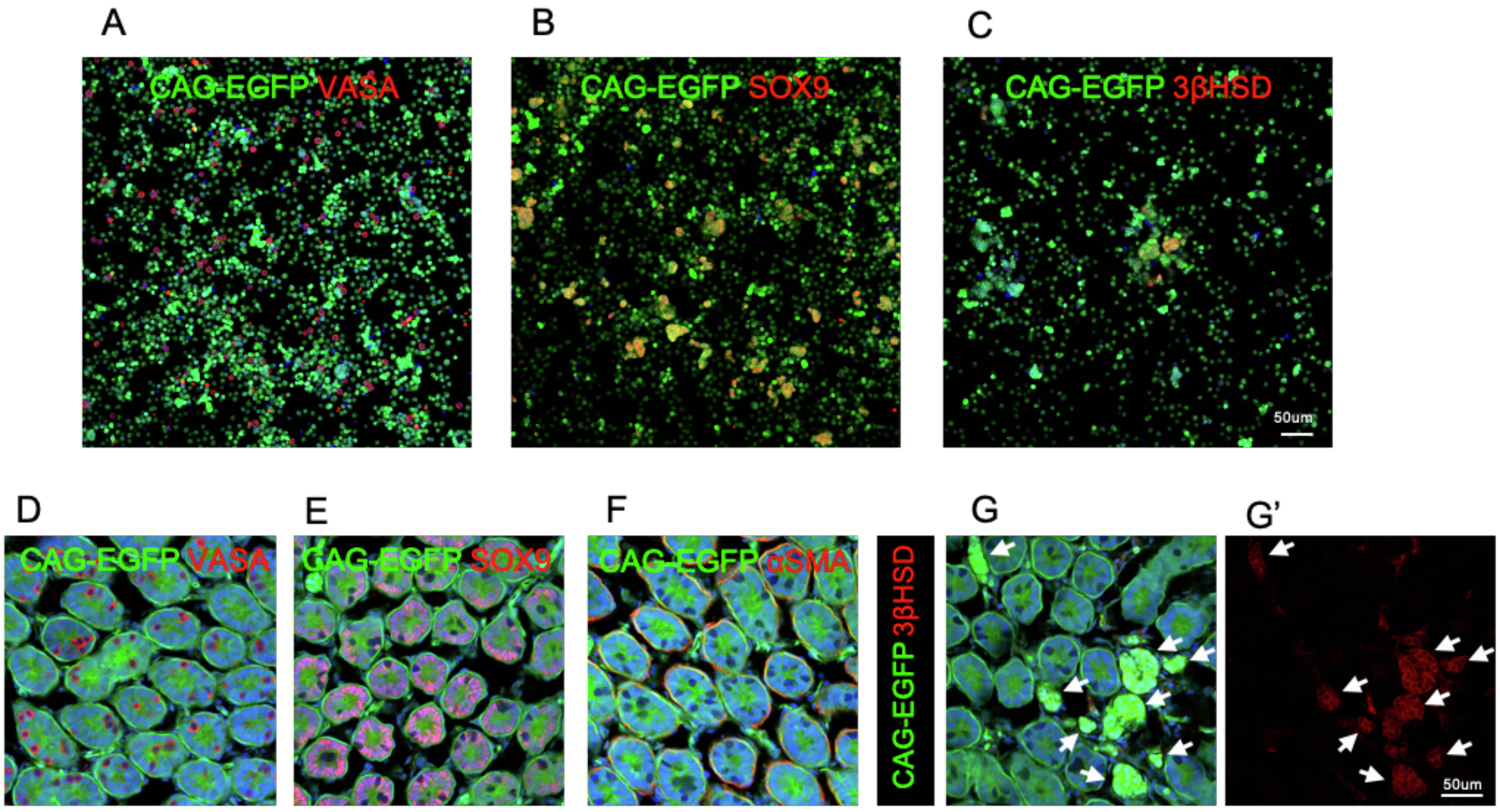

(A–C) Dissociated 5.5 dpp rat testicular cells showing VASA^{+} germ cells, SOX9^{+} Sertoli cells, and 3βHSD^{+} Leydig cells labeled with anti-GFP antibody and counterstained with Hoechst (blue). (A) Donor spermatogonia were positive for VASA (red). (B) Donor Sertoli cells were labeled with SOX9 (red). (C) Donor Leydig cells were identified using anti-3βHSD antibody (red). (D–G') Immunofluorescent staining of testis sections from 6.5 dpp CAG-EGFP rats. GFP expression (green) co-localized with (D) VASA (red, germ cells), (E) SOX9 (red, Sertoli cells), and (F) αSMA (red, peritubular myoid and vascular endothelial cells). (G and G') Donor Leydig cells (arrows) expressing 3βHSD (red) were also observed. At this developmental stage, donor spermatogenesis had not yet initiated. Donor Leydig cells were underrepresented in the dissociated cell suspensions because of their poor recovery during cell preparation. The tissue-section images are representative of two biologically independent experiments, and the dissociated-cell analyses represent four biologically independent experiments.

Figure S3. Rat spermatid differentiation in xenogeneic seminiferous tubule–like structures within BBC-treated mouse testes.

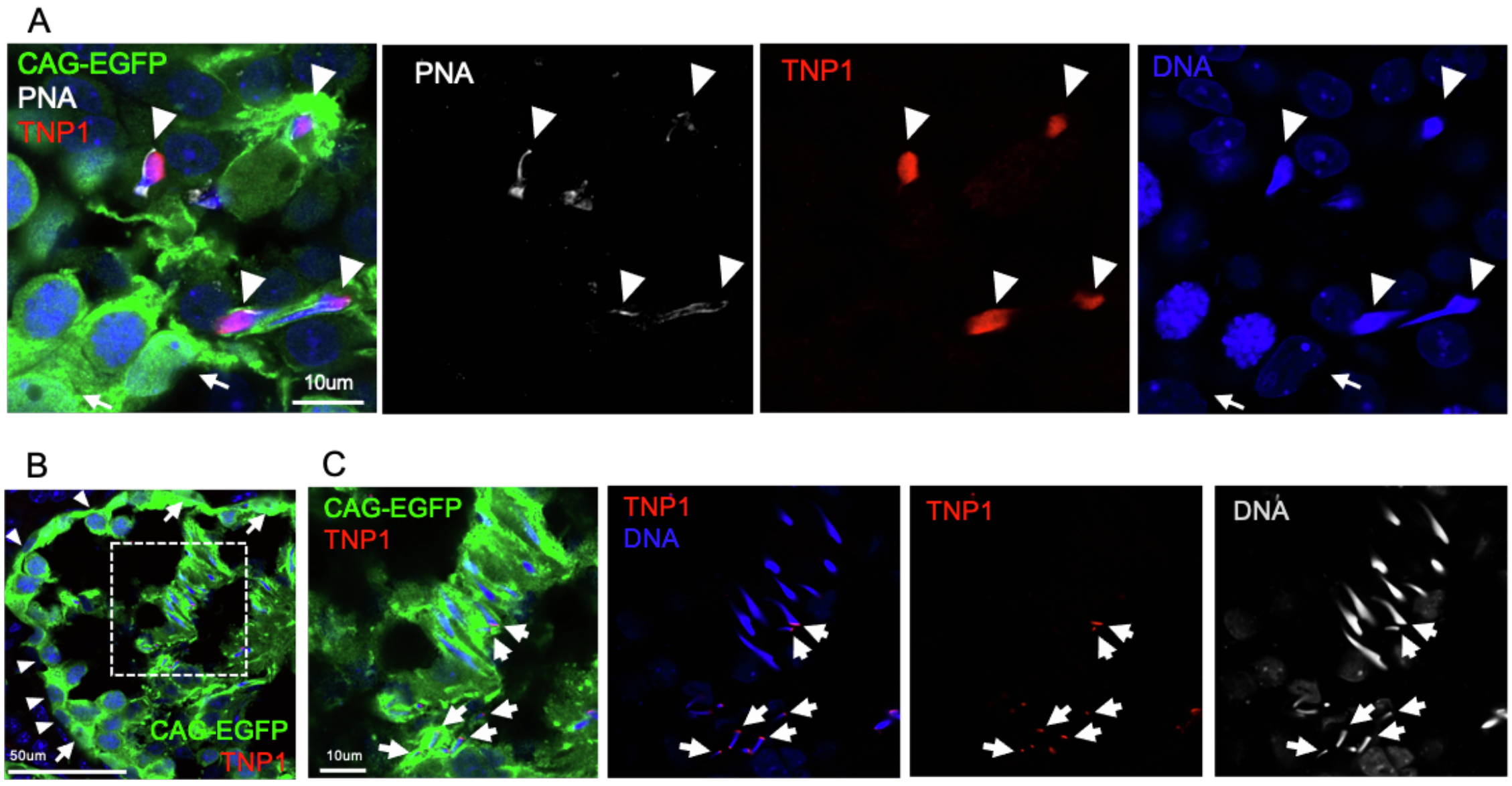

(A) Elongating spermatids at step 12 (arrowheads) showing nuclear-wide expression of TNP1. Rat-derived Sertoli cells (arrows). CAG-EGFP (green), PNA (gray), and TNP1 (red). (B and C) Reconstructed rat seminiferous tubule–like structures. CAG-EGFP (green) and TNP1 (red). (B) Rat-derived Sertoli cells (arrows) and rat-derived spermatogonia (arrowheads). (C) Higher magnification of the dashed region within the lumen of the xenogeneic seminiferous tubule shown in (B). Spermatid nuclei are densely stained, with condensed TNP1, consistent with elongating spermatids at steps 14–15 (arrow). The representative images were selected from multiple transplanted testes among the 15 testes analyzed from 13 recipient mice across five independent transplantation experiments.

Figure S4. Partial reconstruction and spermatogenesis in Busulfan-treated recipient mice following xenogeneic transplantation.

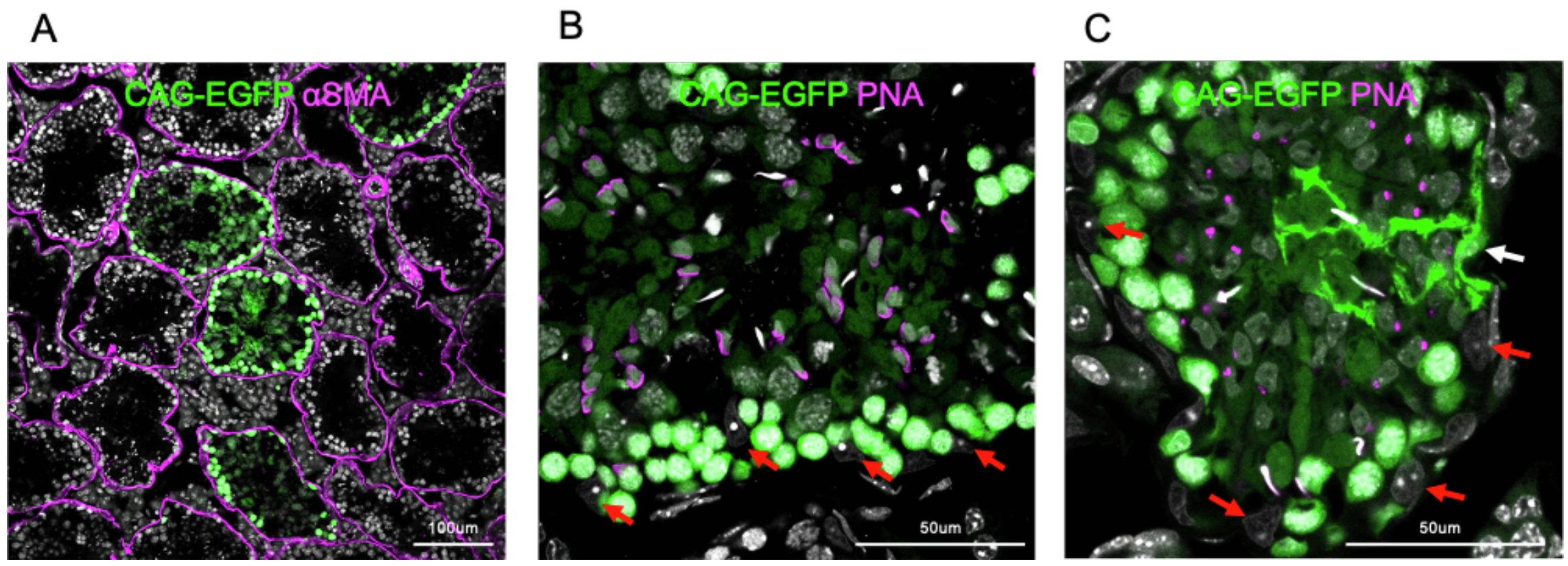

(A and B) GFP⁺ donor-derived rat spermatogonia colonizing seminiferous tubules and undergoing spermatogenic differentiation in association with host Sertoli cells. CAG-EGFP (green) labels all donor-derived cells, and nuclei were counterstained with Hoechst (gray). (A) αSMA-positive peritubular myoid cells are shown in magenta. (B) Host mouse Sertoli cells are indicated by red arrows, and PNA-positive spermatids are shown in magenta. (C) Partial reconstruction of a seminiferous tubule containing donor-derived rat Sertoli cells, host mouse Sertoli cells, and rat germ cells undergoing spermatogenic differentiation. Donor-derived rat Sertoli cells and host mouse Sertoli cells are indicated by white and red arrows, respectively. PNA-positive spermatids are shown in magenta. The representative images in (A–C) were obtained from a single transplanted testis selected from 12 testes examined from six Bus-treated recipient mice across five independent transplantation experiments.
